# Analysis of key factors and equity in influenza vaccination among Chinese adults-evidence from a large national cross-sectional survey

**DOI:** 10.3389/fpubh.2025.1601577

**Published:** 2025-06-05

**Authors:** Bo Dong, Hengxuan Xu, Ning Tang, Menghan Jiang, Zhihao Lei, Yue Han

**Affiliations:** ^1^School Public Health, Zhejiang Chinese Medicine University, Hangzhou, China; ^2^Zhongnan Hospital of Wuhan University, Wuhan, China; ^3^Faculty of Humanities and Social Sciences, Macau Polytechnic University, Macau, China; ^4^School of Humanities and Management, Zhejiang Chinese Medical University, Hangzhou, China; ^5^School of Mathematics, University of Edinburgh, Edinburgh, United Kingdom; ^6^Department of Biostatistics, Brown University, Providence, RI, United States; ^7^The 960th Hospital of the PLA Joint Logistics Support Force, Jinan, Shandong, China

**Keywords:** adults, influenza, vaccination, key factors, equity

## Abstract

**Introduction:**

Influenza is prevalent globally, leading to severe morbidity and mortality. Vaccination remains a critical strategy for influenza prevention. Although previous studies in China have primarily focused on influenza vaccination among children, limited research has addressed the key determinants and equity issues concerning adult influenza vaccination.The purpose of this study was to investigate the key factors influencing influenza vaccination and its equity among Chinese adults.

**Methods:**

The study uses data from the 2021 Chinese General Social Survey (CGSS) (N = 2695).Initially, differences in influenza vaccination among adults with various baseline characteristics were analyzed using the chi-square test. Subsequently, the importance of influencing factors was assessed through a random forest model, with LASSO used for variable selection. Following this, weighted logistic regression analysis was applied to quantify the significant influencing factors. Finally, the concentration index was employed to identify and determine the contribution of important variables to influenza vaccination.

**Results:**

The influenza vaccination rate among Chinese adults is low (6.75%). Key factors identified as influencing adult vaccination include government trust, physician trust, income, aging concerns, health insurance, age, education, and health status. These factors not only have independent effects but also interact to influence vaccination behavior. Regarding individual effects, government trust, physician trust, income, and aging concerns showed positive associations with vaccination rates. Conversely, health insurance status, age, educational attainment, and health status demonstrated negative associations. Regarding the interaction terms, there were positive associations between health insurance and government trust, education and government trust, health and physician trust, government trust, as well as education level and age with the target variables. In contrast, interactions between income and health insurance, as well as income and physician trust negatively influenced vaccination rates. The concentration index for adult influenza vaccination was 0.092. There was inequity in vaccination, with the distribution of vaccinations being skewed toward higher-income individuals. Decomposition analysis further revealed that the primary contributors to vaccination inequity, in descending order of magnitude, were income (32.6%), government trust (9.1%), education (8.7%), age (8.2%), and aging concerns (2.6%).

**Discussion:**

This is the first study to leverage a large micro-survey database in China to analyze the key factors affecting adult influenza vaccination and its equity. By providing new evidence on influenza vaccination among Chinese adults, the findings may inform the optimization of adult immunization policies. To further increase influenza vaccination rates and promote equity among Chinese adults, future policy improvements could consider emphasizing the role of trust in vaccination uptake, subsidizing vaccination costs, and fully utilizing comprehensive intervention strategies to enhance adult influenza vaccination coverage and equity.

## 1 Introduction

Influenza (flu) is a highly contagious respiratory infectious disease caused by influenza viruses. It spreads rapidly and widely through various transmission routes (1). Symptoms are typically severe, including high fever, headache, muscle pain, and general malaise, and the disease often leads to serious complications such as pneumonia and myocarditis (2). Influenza has been classified by the World Health Organization (WHO) as a priority infectious disease for global surveillance. Influenza not only affects the life and health of the public, but also can impose a greater burden on socioeconomic development (3, 4). According to WHO estimates, influenza causes approximately 1 billion infections, 3–5 million severe cases, and 290,000–650,000 respiratory-related deaths annually worldwide (5, 6). In China, influenza is associated with approximately 88,100 respiratory-related deaths each year (7). Vaccination remains the most effective measure for preventing influenza, significantly reducing influenza-related complications, hospitalizations, and deaths (8, 9). At the same time, influenza vaccination is also cost-effective, offering benefits in direct healthcare cost savings and broader public health improvements (10–13). A study in the United States indicated that a 5% increase in vaccination rates could result in 785,000 fewer influenza cases and 11,000 fewer hospitalizations annually (14). Conversely, a decrease in influenza vaccination rates can also bring about a decrease in vaccine protection, increasing the risk of influenza and related complications, particularly in high-risk groups such as infants, young children, the older adult, and patients with chronic diseases (15). Wei et al. analyzed the protective effect of free seasonal influenza vaccination among primary and secondary school students in Fangshan District, Beijing, China, during the 2022–2024 influenza seasons. It is found that the protective effect of the vaccine was 53.41% (95% CI:22.21–72.21%) before adjustment, and 59.80% (95% CI:22.71–77.71%) after adjustment, concluding that the effect of influenza vaccination for primary and secondary school students is considered to be better (16). Li et al. analyzed the effect of influenza vaccination provided free of charge to the older adult population over 65 years in Kaifu District, Changsha City, China. They reported benefits of RMB 130.08, RMB 901.27, and RMB 1,209.19 within the 1st, 3rd, and 6th months of influenza vaccination, with benefit–cost ratios of 1.40:1, 9.73:1, and 13.06:1, respectively. Through the study on the group of the vaccinated group versus the comparison group, it was found that the effect of influenza vaccination was better than that of the comparison group. Studies found that influenza vaccination can reduce the incidence of colds, respiratory and cardiovascular diseases, and improve the health of the older adult population (17).

Influenza vaccination is considered the most critical primary health intervention for controlling influenza epidemics (18). Although many countries have implemented policies aimed at increasing vaccination coverage and equitable access, limited healthcare funding often restricts vaccination availability across entire populations (19, 20). In China, influenza vaccination is not prioritized in public health. In most places, it is not included in the immunization program, and only specific regions, such as Beijing, Zhejiang, and Shenzhen, have implemented “influenza public funding programs” for high-risk groups (schoolchildren and the older adult) or incorporated vaccination costs into social health insurance reimbursement schemes (21). Therefore, in most regions of China, influenza vaccination is generally not reimbursed by the government. Vaccinees must pay for the vaccination themselves, which hinders the coverage and equity of influenza vaccination. Results suggest that influenza vaccination rates and willingness to vaccinate in China remain notably low. According to the Chinese Center for Disease Control and Prevention (CDC), the overall population vaccination rate for the 2022–2023 influenza season is only 3.84%, a rate that is much lower than that of developed countries (82.3% in England, 90.2% in Scotland, 75.2% in the United States 55.0% in Russia, and 56.8% in France) (22, 23). In addition, China’s vaccination service system predominantly emphasizes child immunization, having established comprehensive vaccination assessment clinic and monitoring systems, while the adult vaccination infrastructure remains relatively underdeveloped (24). As a result, adult vaccination rates are particularly low. Evaluations of influenza vaccination for different populations in China showed that influenza vaccination rates were significantly higher in children than in adults (16.66% > 12.75%) (25, 26). Therefore, further analyzing the key factors affecting influenza vaccination in adults and identifying the impact of different factors on vaccination equity are essential for prioritizing strategies to enhance vaccination rates in the future.

Regarding the influenza vaccination of different populations in China, relevant studies can be summarized in the following three aspects. First, regarding the current status of influenza vaccination, relevant studies focus on analyzing the influenza vaccination rate in limited geographic areas or special populations. For example, Zou et al. surveyed 2,261 secondary school students in four Chinese cities and found that 44.7% expressed vaccine hesitancy (27). Hou et al. conducted a cross-sectional survey of 3,849 older adult individuals across ten Chinese provinces, reporting that 37.18% showed vaccine hesitancy (28). Similarly, Tu et al. reported a low influenza vaccination rate (19.37%) among medical staff in urban areas of Nanchang City, highlighting a need to improve vaccination willingness (29). Second, studies on influencing factors of influenza vaccination indicate that marital status, age, household registration, income, education, occupation, vaccination history, health status, chronic disease status, and medical insurance are important factors for vaccination (30–32). Third, studies evaluating the effect of influenza vaccination include a comprehensive meta-analysis assessing vaccine effectiveness (VE) across mainland China. Results showed that VE was 45% (95% CI:18–64%) for children aged 6–35 months who received one dose of influenza vaccine, and 57% (95% CI:50–64%) for those who received two doses of influenza vaccine; for ≥60 years old adults, VE was 18% (95%CI:0–33%); for hospitalized patients, VE was 21% (95% CI:11–44%), influenza vaccine was moderately effective, and VE was higher in children than in the older adult (33).

Regarding vaccine equity, current relevant research focuses on the fairness of vaccine distribution, optimization of vaccine allocation, and enhancement of vaccine supply efficiency through scientific decision-making. For example, studies by WHO and other researchers have highlighted significant disparities in global vaccine supply, with higher vaccine accessibility in high-income countries, and insufficient and poorer vaccine accessibility in low- and middle-income countries. These findings emphasize the necessity of balanced vaccine distribution across regions to achieve the equity goals outlined in the Immunization Agenda 2030 (34–37). In terms of scientific decision-making to optimize vaccine allocation, a study by Shahrooz et al. proposed a decision support system (DSS) that integrates GIS, analytics, and simulation methods to help develop priority-based COVID-19 vaccine allocation scenarios in large urban settings (38). As for the equity of vaccination, the relevant research results from abroad are more abundant, the study by Adam et al. assessed the difference in influenza vaccination between US veterans and non-veterans during 2019–2020 (39). Similarly, Anne et al. analyzed the pneumococcal, polio, and measles vaccines in Ethiopia and explored the impact of socioeconomic, geographic, maternal and child characteristics on vaccination equity (40). In China, only a few studies have analyzed the equity of adult hepatitis B vaccination in rural areas of China (41). Currently, no studies have specifically investigated equity in influenza vaccination among adults.

In terms of research methodology, relevant studies have not only used logistic regression to explore the influencing factors of influenza vaccination in different populations, but also used machine learning methods to address vaccination issues across different demographic groups in China. For example, Wang et al. used logistic regression and decision tree modeling to explore mothers’ willingness to vaccinate their daughters with the HPV vaccine and their influencing factors. The results indicated that integrating these two methods offers a more comprehensive analysis of the complex interrelationships among various determinants, effectively clarifying the specific mechanisms through which each factor influences vaccination willingness (42). Similarly, Qin et al. applied random forest modeling to explore differences in HPV vaccine perceptions among urban and rural middle school girls in Yiyang City, Hunan Province. Employing such machine learning methods not only provides new methods for deepening vaccination-related research from a theoretical perspective, but also provides robust evidence support for optimizing vaccination policies for different populations and improving vaccination levels from a practical level (43).

From the above studies, it can be seen that related scholars have paid extensive attention to influenza vaccination. However, there remain several areas for further exploration. First, current research perspectives tend to be limited, and most studies use surveys to analyze the vaccination rate in specific regions or populations, which limits the sample size and representativeness of the results. Second, the content of existing research is somewhat homogeneous, primarily focusing on vaccination coverage rates and influencing factors, with limited attention given to vaccination equity, particularly lacking empirical evidence regarding influenza vaccination equity in China. Finally, research methodologies need further diversification. Although logistic regression models have been widely used to assess the impact of various factors on vaccination uptake, there remains insufficient exploration of the relative importance of these factors. Therefore, this study takes Chinese adult influenza vaccination as an example and uses representative national survey data (Chinese General Social Survey) to explore the key influencing factors of adult influenza vaccination and its equity. This study specifically includes three objectives: first is to understand the current status of influenza vaccination among the adult population in China; and then to explore the key influencing factors of influenza vaccination among the adult population; and the final aim is to analyze the fairness of influenza vaccination among adults and the contribution of different factors to vaccination equity.

Compared to existing research, this study offers several key contributions. First, by utilizing representative large-scale cross-sectional survey data from China, this research extends its analysis to encompass all adults, enhancing the generalizability of the findings. Second, a random forest model is employed to assess the relative importance of various factors influencing adult influenza vaccination, enabling the identification of critical determinants and facilitating a more precise evaluation. Third, the study examines equity in adult influenza vaccination, quantifying the contributions of key determinants to vaccination inequity. This not only enrich the existing analyses of vaccination equity but also provide more precise interventions to improve the current status of adult influenza vaccination. The results are expected to offer valuable insights into potential target populations and thus provide a scientific basis for optimizing future influenza vaccination strategies for adults.

## 2 Methods

### 2.1 Data sources

The data used in this study are derived from the 2021 Chinese General Social Survey (CGSS), a large-scale, continuous random sample survey project that began in 2003 by the China Center for Survey and Data of Renmin University of China. As China’s earliest comprehensive, nationwide academic survey, the CGSS is widely recognized for its authoritative and extensive database, characterized by large sample sizes, wide geographical coverage, and diverse content (44).

The survey boasts a comprehensive sample covering 19 provinces, autonomous regions, and municipalities directly under the central government (45). It employs a multi-stage stratified sampling approach, with counties serving as primary sampling units. Post-stratification weights were applied to adjust for oversampling, thereby ensuring the survey results accurately represent the general population in China (46). The multi-stage stratified sampling method of the survey was implemented as follows: the first stage was stratification and sample frame construction. According to the administrative division of China, the whole country is divided into several provincial units (provinces, autonomous regions, and municipalities directly under the central government). Within each provincial unit, further stratification occurred based on urban–rural status, economic development level, and other relevant criteria to construct the sampling framework. The second stage is the main sampling unit extraction. In each stratum, a certain number of county-level administrative units or streets and townships are randomly selected using a multi-stage stratified probability proportional sampling (PPS) method according to population size or other relevant indicators. The third stage is subsampling unit extraction. In each selected primary sampling unit, several sub-sampling units of communities, villages, or neighborhood committees are randomly selected to ensure coverage of different socio-economic backgrounds. The fourth stage was household and individual sampling. In each selected sub-sampling cell, several households were selected using a randomization method. Within each sampled household, one individual who met the survey criteria was randomly selected as a respondent. The survey also used enumerator training, on-site supervision, and data validation to ensure the authenticity and integrity of the data.

**Figure 1:**
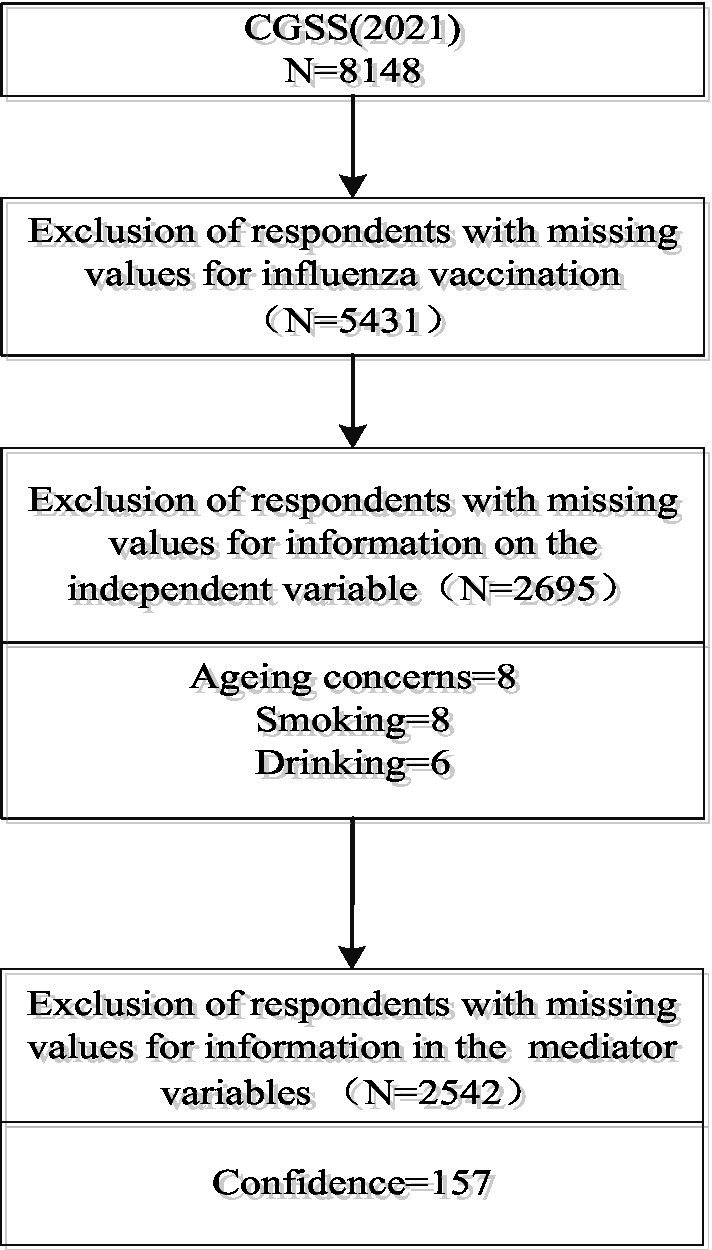
Flow chart of sample inclusion and exclusion.

The 2021 CGSS targets Chinese citizens aged 18 years and older, systematically and comprehensively collecting data at social, community, household, and individual levels. Key areas of data collection include demographics, household characteristics, labor and employment status, social attitudes, and lifestyle practices. Notably, the 2021 survey includes a new section on COVID-19 vaccination status, which documents information on COVID-19 vaccination, pandemic influences on behaviors and attitudes, individual perceptions of vaccination, and many other core factors needed for the study. This is a wealth of information that has not been presented in other similar surveys, and provides a wealth of data to support the exploration of vaccine hesitancy. The 2021 survey collected a total of 8,148 valid samples nationwide. After processing for missing sample values, 2,695 respondents were included in the analysis of the key influencing factors and fairness. 2,542 respondents were included in the analysis of mediating mechanisms, and the sample inclusion and exclusion process is shown in Figure 1.

### 2.2 Variables

#### 2.2.1 Dependent variable

The dependent variable in this study is influenza vaccination. In the CGSS2021 questionnaire, the specific question about dependent variables was “Have you had a flu vaccine in the last year?” Respondents could answer either “vaccinated” and “not vaccinated.” Based on responses, a dummy variable was constructed to measure whether the respondents had received the flu vaccine or not. If the answer was “yes,” it is defined to receive the corresponding flu vaccine, otherwise they had not, i.e., vaccinated = 1; not vaccinated = 0.

#### 2.2.2 Independent variables

Individual vaccination behavior may be affected by other factors. Based on existing relevant studies and considering data availability (47, 48), we controlled for a comprehensive set of variables in the empirical models to avoid potential omitted variable bias. The control variables included 23 factors such as gender, age, income, religion, education, etc., and the specific meaning and definition of each variable are shown in Table 1.

**Table 1:** Variable selection and definitions.

Variables		Definitions
Dependent variables	Influenza vaccination	No vaccinated = 0; Vaccinated = 1
Independent variables	Age	≤29 = 1; 30–59 = 2; ≥ 60 = 3
	Gender	Male = 1; Female = 2
	Income	<20,000 = 1; 20,000− = 2; 50,000− = 3; 100,000− = 4
	Religious belief	No religion = 0; Religion = 1
	Education	Less than high school = 1 High school = 2 University = 3
	Work	Without work = 1; With work 2
	Health	Unhealthy = 1; Basically healthy = 2; Healthy = 3
	Marriage	Unmarried = 1; Married = 2
	Household registration	Rural = 1; Urban = 2
	Nationality	Han minorities = 1; Ethnic minorities = 2
	Mobile state	Non-mobile population = 1; Mobile population = 2
	Region	West = 1; Central = 2; East = 3
	Health insurance	Not participating = 0; Participating = 1
	Aging concerns	Completely disagree = 1; Disagree = 2; Neither agree nor disagree = 3 Agree = 4; Completely agree = 5
	Chronic disease	No = 0; Yes = 1
	Smoking	No = 0; Yes = 1
	Drinking	No = 0; Yes = 1
	No coronavirus vaccination	No vaccinated = 0; Vaccinated = 1
	Government trust	Decreased = 1; Largely unchanged = 2; Increased = 3
	Medical staff trust	Decreased = 1; Largely unchanged = 2; Increased = 3

### 2.3 Statistical methods

#### 2.3.1 Random forest model

Random forest model is an integrated machine learning algorithm based on decision trees, recognized for its clear interpretability, structural clarity, and stability in evaluating factor importance. It not only compares the importance of each variable and identifies the relatively important variables, but also has high accuracy, high testing efficiency, good stability, and produces more reliable results (49). Compared with other machine learning models, the random forest model has the advantages of visualizing feature importance, less adjustment, reducing the risk of overfitting, and capturing complex interactions between features. This approach is widely applied in medical and public health research (50–52). Therefore, based on similar studies, this study also used the random forest model to measure the importance of factors influencing influenza vaccination among Chinese adults.

Random forest algorithm consists of multiple decision trees generated through bagging, a process that randomly samples subsets of data to create multiple training sets. Each training set is analyzed using a decision tree as a base classifier. The final prediction is made by aggregating the outcomes from these trees through majority voting. This algorithm can perform classification, regression, and prediction tasks effectively. Compared to a single decision tree algorithm, random forests exhibit better accuracy and prediction performance and are less susceptible to overfitting. Importance analysis is to use the best variables selected in the decision tree as classification nodes, to rank the importance of variables (53). The specific analysis steps are as follows: ① For each decision tree, select the corresponding out of bag data (out of bag, OOB) to calculate the out of bag data error, recorded as err OOB1; ② Randomly add the noise interference to the feature X of all the samples of the OOB (you can randomly change the value of the samples in the feature X), and calculate the out of bag data error again, recorded as err OOB2; ③ Given that there are *N* trees in the forest, the importance of feature X is calculated using the formula (53).

$$\mathrm{OOB}\_\text{store}=\frac{\sum_{i=1}^{N}(\text{erroob}2i-\text{erroob}1i)}{N}$$

This metric indicates the importance of a feature because if adding random noise significantly increases the OOB error (i.e., err OOB2 rises), it indicates that the feature has a significant impact on the prediction results of the sample, thereby demonstrating higher importance.

#### 2.3.2 Concentration index decomposition

In studies related to public health, methods for measuring inequality usually include the Concentration Index (CI), the Gini coefficient, the Oaxaca-Blinder decomposition, the Lorenz curve, and the method of extreme variance (54, 55). Among these, the Concentration Index has the advantages of quantifying the tendency of health service utilization or health inequality, revealing structural inequality, identifying the attributing factors of inequality through decomposition techniques, and not being affected by the absolute level of research indicators (56–58). Currently, the concentration index is widely used to measure income-related inequalities in health (59, 60). Based on the above analysis, this study also used the concentration index to analyze the equity of influenza vaccination among Chinese adults as well as to measure the extent to which the relevant influencing factors contribute to the equity of vaccination. The CI is calculated using the following formula:

$$\mathrm{CI}=2\mathrm{cov}(y_{i}\;R_{i})/\mu$$

Where $y_{i}$ represents the outcome variable vaccination, μ is the average of the variable in the population, and $R_{i}$ represents the fractional rank of sample i in the income distribution. The value of CI ranges from (−1, 1), CI > 0 indicates that there is a pro-rich inequality in the outcome variable, and CI < 0 indicates that there is a pro-poor inequality in the outcome variable. A larger absolute value of CI indicates greater sensitivity of the outcome distribution to income level and a higher degree of inequality.

This study uses the concentration index decomposition method proposed by WAGSTAFF (61) to decompose the factors that may affect the fairness of adult vaccination, and utilizes the degree of contribution of different factors to fairness after decomposition to rank them, to clarify the main source factors of inequity, and then to control or eliminate them in a targeted manner. The specific decomposition formula is as follows:

$$C=\sum_{j}(\beta_{j}{\bar{X}}_{j}/\mu )\;\;C_{j}+{\mathrm{GC}}_{\varepsilon }/\mu$$

C is the unstandardized concentration index, $\beta_{j}$ , ${\bar{X}}_{j}$ , and $C_{j}$ are the regression coefficients (replaced by marginal effects), means, and concentration indices of influences j , respectively, $\beta_{j}{\bar{X}}_{j}/\mu$ indicates the magnitude of the contribution of influences j to inequality in vaccination, ${\mathrm{GC}}_{\varepsilon }$ is the concentration index of the residual term, and μ is the mean of the vaccination utilization outcome (i.e., the dependent variable).

### 2.4 Statistical analysis

**Figure 2:**
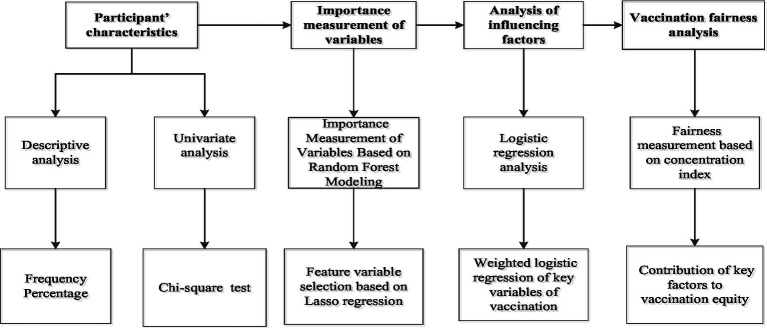
The flow chart of the study.

The Stata 22.0 and RStudio software were used to analyze the data. Count data were expressed as counts and percentages (%). The chi-square test was used for univariate analysis to assess group differences. Variables with statistically significant differences in the univariate analysis were subsequently included in the random forest model, which was implemented using RStudio to compute and rank the importance scores of each variable. Following this, LASSO analysis was used for the selection of the variables, and the screened variables were analyzed by applying the weighted logistic regression analysis was used to analyze the selected variables. Finally, the decomposition of the vaccination concentration index was used to identify the contribution of important variables to influenza vaccination. The flow chart is shown in Figure 2.

## 3 Results

### 3.1 Respondents characteristics

Table 2 shows the results of descriptive statistics of the main variables in this study. Among the 2,695 adult respondents, 6.75% had received the influenza vaccine, while 93.25% had not. In terms of age distribution, 70.98% of respondents were under 50 years old, and 29.02% were aged 60 or older. Regarding income, 61.64% of respondents were classified as having relatively low incomes. Education levels were generally low: only 20.45% of respondents had attained college-level education or higher, while 79.65% had completed high school or less. Health status was reported to be relatively good, with 67.77% of respondents describing themselves as healthy and 23.34% as basically healthy. Health insurance coverage was high, with 86.31% of respondents having health insurance. Concerning aging-related concerns, 66.60% of respondents expressed high concern, 11.65% moderate concern, and 21.57% low concern. Regarding chronic disease status, 62.15% of respondents reported having no chronic illness. As for trust metrics, 87.34% of respondents reported increased trust in the government, and 84.94% expressed increased trust in healthcare professionals. Regarding differences in influenza vaccination among adults with different baseline characteristics, there were statistically significant differences in vaccination between age, gender, income, education, health status, health insurance, aging concerns, chronic disease status, trust in government, and trust in medical personnel.

**Table 2:** Basic characteristics of respondents.

Variables		Total		No vaccinated		Vaccinated		χ^2^	*p*
		*n*	%	*n*	%	*n*	%		
Age	≤29	563	20.89	511	20.33	52	28.57	7.140	0.028
	30–59	1,350	50.09	1,270	50.54	80	43.96		
	≥60	782	29.02	732	29.13	50	27.47		
Gender	Male	1,221	45.31	1,150	45.76	71	39.01	3.121	0.077
	Female	1,474	54.69	1,363	54.24	111	60.99		
Marriage	Unmarried	761	28.24	713	28.37	48	26.37	0.335	0.563
	Married	1934	71.76	1800	71.63	134	73.63		
Nationality	Han minorities	2,485	92.21	2,312	92.00	173	95.05	2.202	0.318
	Ethnic minorities	210	7.79	201	8.00	9	4.95		
Income	<20,000	844	31.32	790	31.44	54	29.67	7.693	0.053
	20,000−	817	30.32	774	30.80	43	23.63		
	50,000−	569	21.11	518	20.61	51	28.02		
	100,000−	465	17.25	431	17.15	34	18.68		
Religious belief	No religion	2,494	92.54	2,321	92.36	173	95.05	1.786	0.181
	Religion	201	7.46	192	7.64	9	4.95		
Education	Less than high school	1,645	61.04	1,546	61.52	99	50.40	7.084	0.029
	High school	499	18.52	452	17.99	47	25.82		
	University	551	20.45	515	20.49	36	19.78		
Work	Without work	1,335	49.54	1,244	49.50	91	50.00	0.017	0.897
	With work	1,360	50.46	1,269	50.50	91	50.00		
Health	Unhealthy	240	8.89	210	8.36	26	14.29	9.112	0.011
	Basically healthy	630	23.34	597	23.76	33	18.13		
	Healthy	1829	67.77	1706	67.89	123	67.58		
Health insurance	Not participating	369	13.69	333	13.25	36	19.78	6.122	0.013
	Participating	2,326	86.31	2,180	86.75	146	80.22		
Mobile state	Non-mobile population	1908	70.80	1774	70.59	134	73.63	0.755	0.385
	Mobile population	787	29.20	739	29.41	48	26.37		
Household registration	Rural	1,578	58.55	1,476	58.73	102	56.06	0.506	0.477
	Urban	1,117	41.45	1,307	41.27	80	43.96		
Region	West	482	17.88	454	18.07	28	15.38	1.754	0.416
	Central	649	24.08	609	24.23	40	21.98		
	East	1,564	58.03	1,450	57.70	114	62.64		
Aging concerns	Completely disagree	95	3.53	84	3.34	11	6.04	10.549	0.032
	Disagree	491	18.22	465	18.50	26	14.29		
	Neither agree nor disagree	314	11.65	283	11.26	31	17.03		
	Agree	1,201	44.56	1,126	44.81	75	41.21		
	Completely agree	594	22.04	555	22.09	39	21.43		
Chronic disease	No	1,675	62.15	1,550	61.68	125	68.68	3.537	0.060
	Yes	1,020	37.85	963	38.32	57	31.32		
Smoking	No	2086	77.40	1944	77.36	142	78.02	0.043	0.836
	Yes	609	22.60	569	22.64	40	21.98		
Drinking	No	1,666	61.82	1,555	61.88	111	60.99	0.057	0.812
	Yes	1,029	38.18	958	38.12	71	39.01		
No coronavirus vaccination	No vaccinated	712	26.42	668	26.58	44	24.18	0.505	0.477
	Vaccinated	1983	73.58	1845	73.42	138	75.82		
Government trust	Decreased	56	2.07	42	1.67	7	3.85	6.675	0.036
	Largely unchanged	286	10.58	273	10.86	13	7.14		
	Increased	2,360	87.34	2,198	87.47	162	89.01		
Medical staff trust	Decreased	101	3.75	87	3.46	14	7.69	8.511	0.014
	Largely unchanged	305	11.32	284	11.30	21	11.54		
	Increased	2,289	84.94	2,142	85.24	146	80.77		

### 3.2 Importance measures of factors influencing influenza vaccination in adults

#### 3.2.1 Random forest data selection

To measure the importance of each factor that influences adult influenza vaccination, a random forest model was constructed using vaccination status as the dependent variable and relevant influencing factors as independent variables. We randomly divided the samples into a training set (80%) and a validation set (20%) and used a random forest classifier for the classification task. To optimize the model performance, randomized search was employed to select the best hyperparameters. Randomized Search CV was performed by randomly selecting the parameter combinations several times, performing a 5-fold cross-validation, and selecting the parameter combinations with the best scores, i.e., the highest accuracy rate, as the final results. The value ranges and interpretations of each parameter are shown in Table 3.

**Table 3:** Analysis of optimal parameter combinations for the random forest model.

Parameters	Interpretation	Range of values	Optimal parameter values
n_estimators(ntree)	The number of trees in the random forest, i.e., how many decision trees are constructed	1–500	180
max_depth	Maximum depth per tree, limiting the depth of the tree	1–20	15
min_samples_split(mtry)	Minimum number of samples required for each internal node split, affecting the complexity of the tree	2–20	10
min_samples_leaf	Minimum number of samples per leaf node, minimum sample size of control leaf node	1–10	6

**Figure 3:**
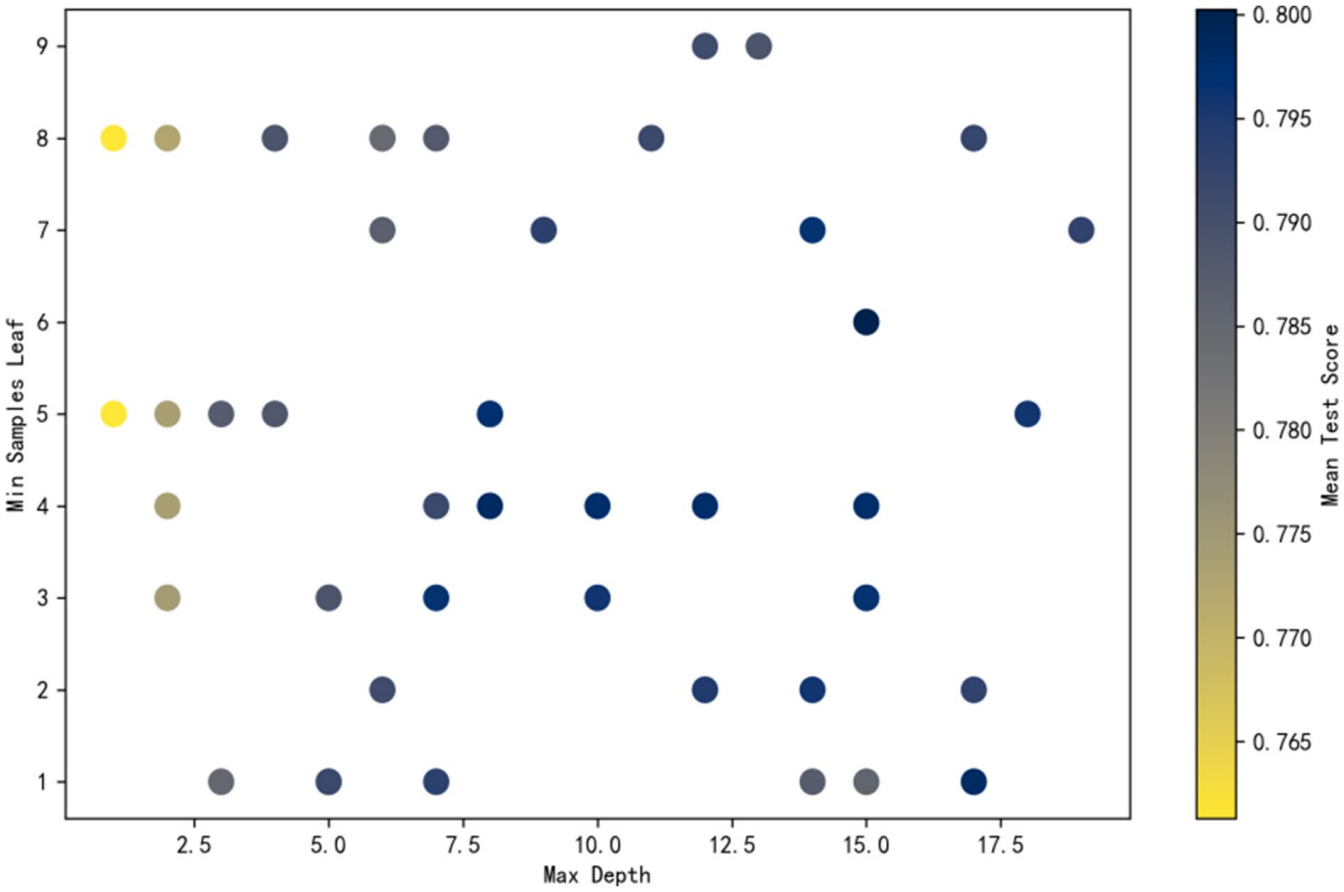
Diagram of random search process.

The process of random search is as follows: considering the large number of parameters, only the search process with the maximum depth as the horizontal axis and the minimum number of leaf node samples as the vertical axis is shown here. Color intensity reflects accuracy, with darker shades indicating higher performance. It can be observed that the point with maximum depth of 15 and minimum number of leaf node samples of 6 has the highest accuracy. This combination was determined to be the optimal hyperparameter setting. The final test set accuracy was 0.8581 (Figure. 3).

#### 3.2.2 Ranking of the importance of influencing factors

**Figure 4:**
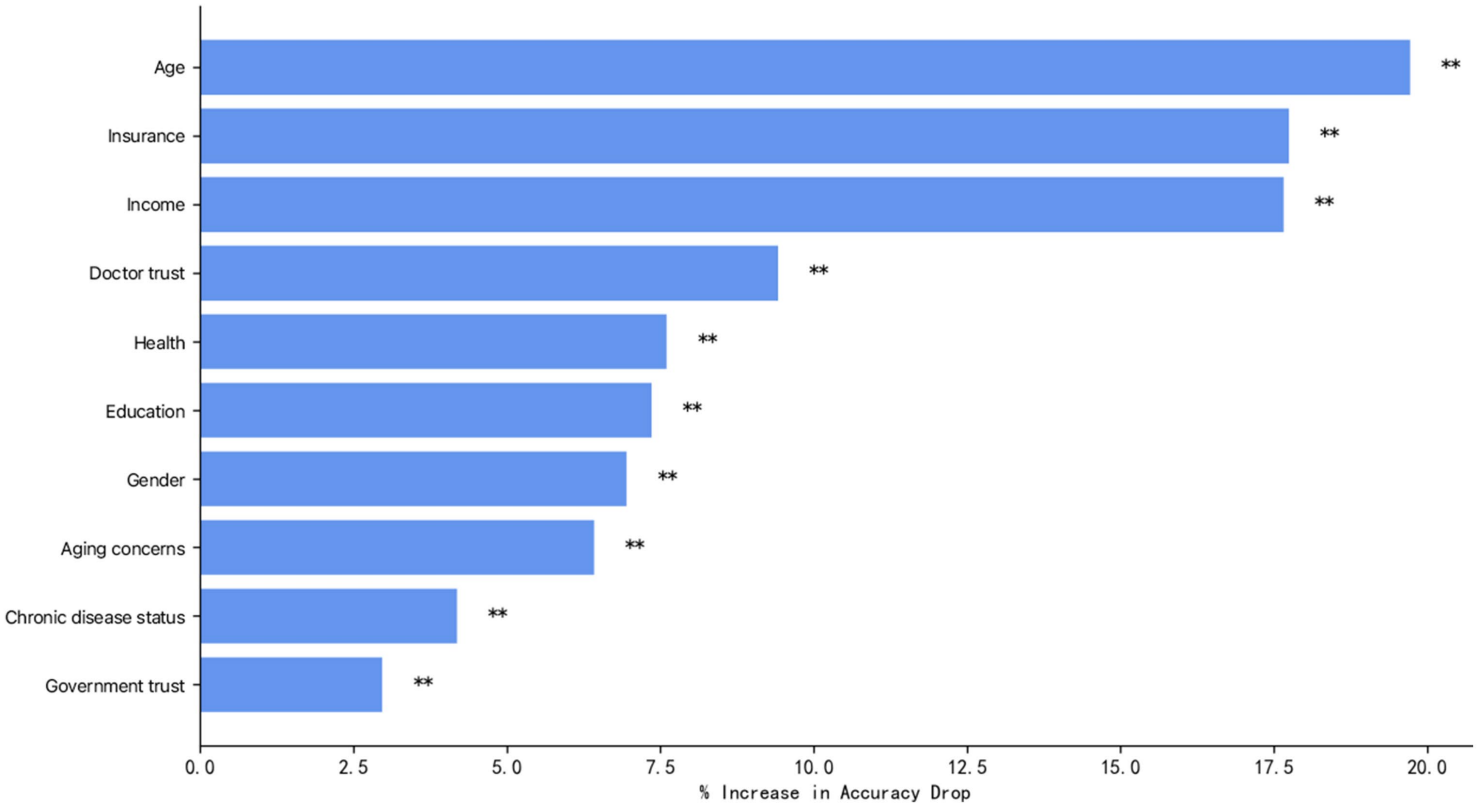
Ranking of importance of variables characterizing influenza vaccination.

Vaccination was used as the dependent variable, and variables with statistically significant differences in the one-way analysis were included in the random forest model. Using the output results of the Random Forest program package in R Studio, the importance ranking of the influencing factors was performed in terms of %Inc. MSE (Mean Reduction in Precision), and the larger the %Inc. MSE, the higher the importance of the variable in the influencing factors (62). Figure 4 demonstrates the results of the importance measure of influencing factors of influenza vaccination in adults. From highest to lowest, the most influential factors were: age, health insurance, income, physician trust, health status, education level, gender, attitude toward aging, chronic disease status, and government trust. The corresponding eigenvalues, 95% CIs, and significance of the variables with different characteristics are shown in Table 4.

**Table 4:** Selection of characteristic variables for influenza vaccination in adults.

Variables	Eigenvalue	95% CI	*p*-values
Aging concerns	6.418	0.012–0.035	2.560E-61
Income	17.654	0.047–0.069	5.446E-97
Education	7.353	0.0126–0.0327	1.872E-65
Age	19.717	0.052–0.080	1.070E-96
Health	7.603	0.015–0.034	1.953E-72
Gender	6.948	0.012–0.029	2.067E-64
Medical staff trust	9.415	0.022–0.042	3.648E-76
Chronic disease	4.182	0.007–0.022	1.219E-58
Government trust	2.968	0.005–0.015	4.997E-62
Health insurance	17.741	0.045–0.074	9.106E-91

*p*-values are calculated by permutation importance permutation importance method; 95% confidence intervals are obtained by Bootstrap method.

#### 3.2.3 Feature variable selection based on LASSO regression

In this section, we first calculate the chi-square statistic and *p*-value of each feature variable over the chi-square test to assess the correlation between each feature and the target variable. A *p*-value less than 0.05 indicates a statistically significant association, meaning that the feature is suitable for inclusion in subsequent model training. Table 5 shows the correlations between different features and the target variables, where education, health, income, age, insurance, aging concerns, government trust and physician trust are the significant feature variables.

**Table 5:** Correlations between different characteristics and target variables.

Feature	Chi^2^ statistic	*p*-value
Aging concerns	21.086	3.044E-04
Income	44.589	1.131E-09
Education	7.509	2.341E-02
Age	111.734	5.462E-25
Health	8.908	1.163E-02
Gender	1.863	1.723E-01
Medical staff trust	102.678	5.056E-23
Chronic disease	1.105	2.932E-01
Government trust	92.410	8.580E-21
Health insurance	144.385	2.927E-33

Secondly, the significant variables are used to generate interaction terms. A LassoCV model with five-fold cross-validation was applied to both main effect and interaction variables, with the maximum number of iterations set to 1,000. By training Lasso regression on the interaction term features, the model selects the features that have a significant effect and shrinks the coefficients of the other insignificant features to zero. This allows LASSO regression to perform both feature selection and dimensionality reduction, effectively filtering out irrelevant variables and mitigating the risks of multicollinearity and overfitting. Compared with the traditional stepwise regression, it can deal with all the independent variables at the same time, which enhances the stability of the model. Currently, this method has been widely used to study related public health issues, including the identification of factors influencing the utilization of health services by mobile populations (63), the analysis of factors influencing per capita health costs (64), and the study of factors influencing the risk of the combined incidence of cardiovascular disease in older adult patients with chronic diseases (65).

**Figure 5:**
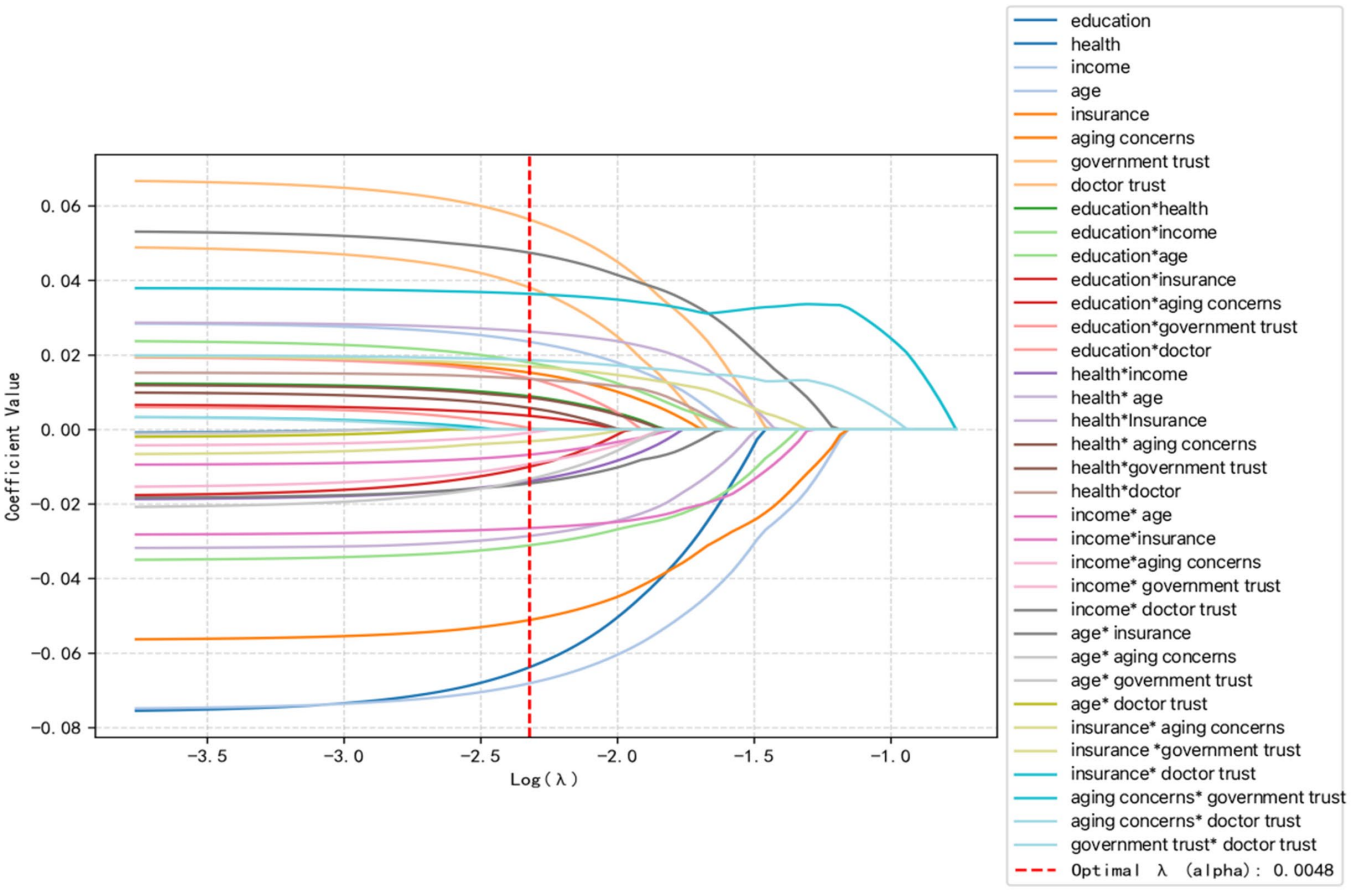
LASSO variable screening plot. Vertical coordinates are the values of the coefficients and the lower horizontal coordinate is log (*λ*).

The paths of the coefficients at different values of *λ* are obtained via LASSO. Path and visualized by assigning different colors to each interaction term feature. Each curve shows how the regression coefficients of each interaction feature change when the regularization strength changes. The optimal λ is 0.0048 (Figure 5). Based on this, the chi-square test was performed again, and the final significant variable chosen was obtained as: education, health, income, age, insurance, aging concerns, government trust, physician trust, education*health, education*income, education*age, education*insurance, education*aging concerns, education*government trust, education*doctor, health*income, health* age, health*Insurance, health* aging concerns, health*government trust, health*doctor, income* age, income*insurance, income*aging concerns, income* government trust, income* physician trust, age* insurance, age* aging concerns, age* government trust, age* physician trust, insurance* aging concerns, insurance *government trust, insurance* physician trust, aging concerns* government trust, aging concerns* physician trust, government trust* physician trust.

#### 3.2.4 Weighted logistic regression analysis of key factors for influenza vaccination in adults

**Figure 6:**
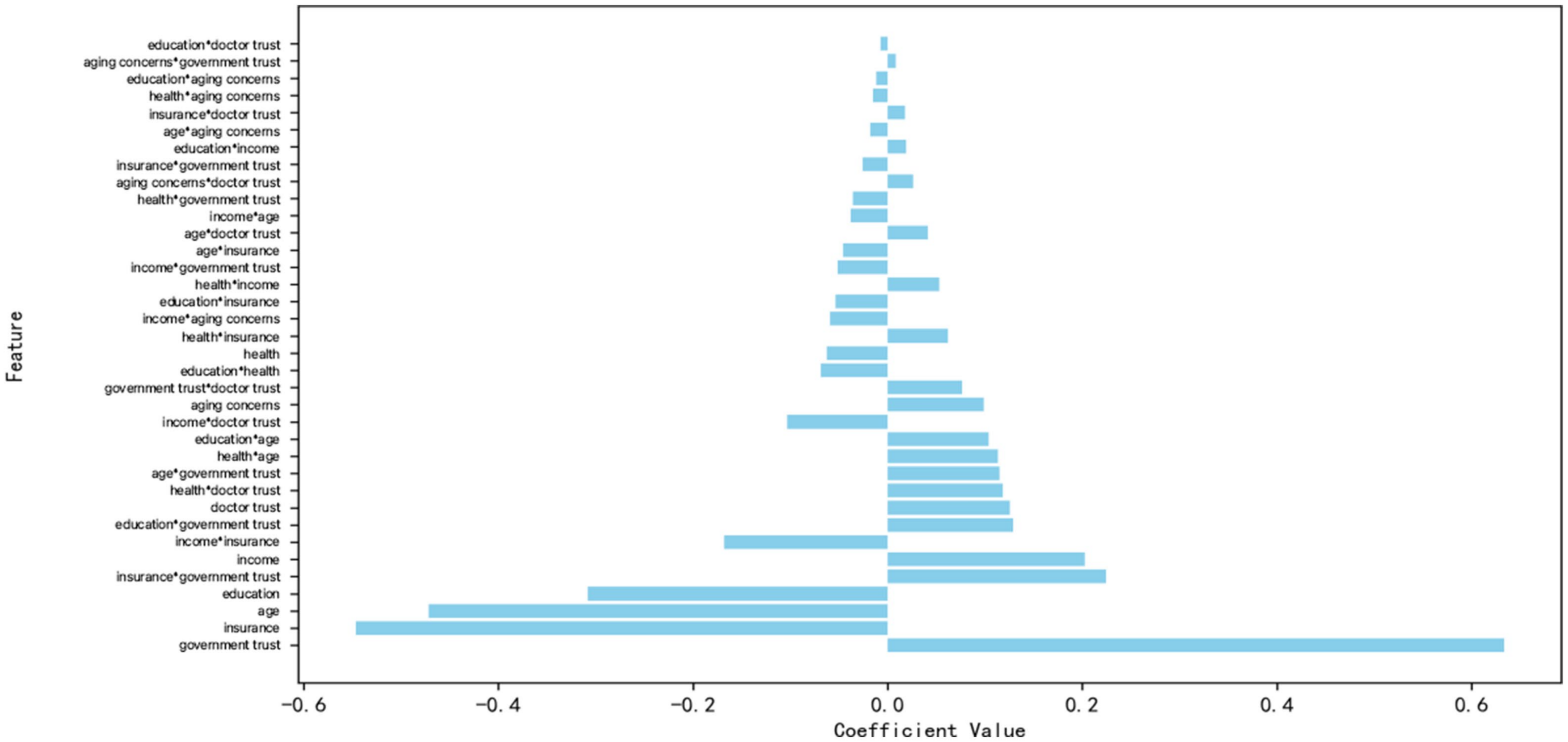
Logistic regression visualization of significant influences on adult influenza vaccination.

Selected salient feature variables were entered into the weighted logistic regression model, and these feature variables were standardized. The model used L2 regularization to prevent overfitting, an optimized Liblinear optimization algorithm suitable for small datasets, and set the maximum number of iterations to 1,000. To deal with the problem of category imbalance, a weighting algorithm was used to compute the sample weights, which automatically adjusts the weights of the categories so that a small number of categories receive more attention. A 5-fold cross-validation was performed by cross_val_score, and the AUC value of the model was evaluated using scoring = “roc_auc”, which ensured that the model’s performance was evaluated on different subsets. After training, the AUC values of the model were calculated using roc_auc_score. Finally, a Hosmer-Lemeshow goodness-of-fit test was performed to further validate the model fit. The final AUC value of 0.8023 obtained in this study indicates that the model classifies well on the entire dataset. The final objective function value = 0.2042, the objective function value indicates the loss function of the model, and a smaller value indicates a better model fit. After 7 iterations, the optimization converged successfully, and the loss value of the model was 0.2042. The *p*-value of the Hosmer-Lemeshow test was 0.057, which is greater than 0.05, showing that the model fits better. Figure 6 demonstrates the results of the logistic regression visualization analysis of the important influencing factors of adult influenza vaccination. The results indicate that not only individual-level variables but also interactions between different features significantly impact vaccination outcomes. In terms of individual characteristic variables, there are positive associations between government trust, physician trust, income, and aging concerns and vaccination, while there are negative associations between health insurance, age, education, and health and vaccination. Regarding the interaction terms, health insurance* government trust, education*government trust, health*physician trust, age*government trust, and education level*age had positive associations with the target variables, while the interaction terms of income*health insurance, and income*physician trust had positive associations with vaccination.

### 3.3 Equity analysis of influenza vaccination for adults

#### 3.3.1 Influenza vaccination equity evaluation

**Figure 7:**
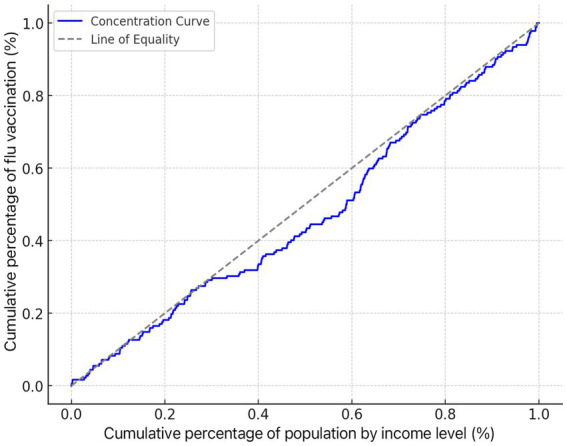
Concentration curve of influenza vaccination in adults.

The concentration index for adult influenza vaccination, CI = 0.092, was greater than 0 and significant (Table 6), and the concentration curve showed a concave trend below the absolute fairness line (Figure 7), indicating that the distribution of adult influenza vaccination was more skewed toward those with higher income levels.

**Table 6:** Results of the concentration index of influenza vaccination in adults.

Methods	95% CI	Standard error	Significance
Delta	0.032–0.208	0.045	0.008***
Bootstrap	0.028–0.215	0.048	0.042*

The Bootstrap method is tested with a sample size of 1,000; *** indicates significant at the 1% level of significance; ** indicates significant at the 5% level of significance; * indicates significant at the 10% level of significance.

#### 3.3.2 Decomposition of the contribution of key factors to equity

Using the eight influencing factors selected in the logistic regression as the independent variables that have a significant impact on adult influenza vaccination, and vaccination as the dependent variable, the study further explored the contribution of these factors to vaccination equity, and the results of the analyses are shown in Table 7. From the results of the decomposition of the concentration indices, the concentration of government trust, age, income, education, and aging concerns on inequality in adult influenza vaccination was positive, indicating that these five factors together contributed to vaccination inequality, of which the higher contributing factors were income (32.6%), income (9.1%), and aging concern, respectively. The concentration indices for health insurance, physician trust, and health on inequality in adult influenza vaccination are all positive, indicating that these five factors work together to contribute to inequality in vaccination, with the factors that contribute to inequality in vaccination to a greater extent being income (32.6%), government trust (9.1%), and educational attainment (8.7%), respectively; whereas the concentration indices for health insurance, physician trust, and health on inequality in adult influenza vaccination are all negative, indicating that these three factors contribute to inequality in vaccination, with the factors that contribute to a greater extent being income (32.6%), trust in government (9.1%), and educational attainment (8.7%). Inequality, with health insurance (39.1%) and physician trust (16.3%) playing a larger role.

**Table 7:** Decomposition of the concentration index for influenza vaccination in adults.

Variables	Coefficient of elasticity	Concentration index	Contribution	Contribution rate (%)
Government trust	0.07	0.12	0.008	9.1
Health insurance	−0.18	−0.20	0.036	39.1
Age	0.05	0.15	0.008	8.2
Education	0.08	0.10	0.008	8.7
Income	0.15	0.20	0.030	32.6
Medical staff trust	−0.15	−0.10	0.015	16.3
Aging concerns	0.03	0.08	0.002	2.6
Health	−0.12	−0.05	0.006	6.5

### 3.4 Further analysis

#### 3.4.1 Mediating mechanisms by which education influences influenza vaccination model fit coefficients

This study identified a negative association between education and influenza vaccination among Chinese adults. To further analyze the intrinsic mechanism by which education influences vaccination, structural equation modeling was used to explore in depth the mediating path between education and vaccination. Regarding the choice of mediating variables, existing studies have pointed out that the psychological antecedents can be described as the psychological states or emotions that people hold during the vaccination program, which can reveal the complex psychological mechanisms behind the phenomenon of vaccine hesitancy (66). Several empirical studies have demonstrated that psychological antecedents serve as key mediators linking external variables to vaccination outcomes. For instance, Maietti et al. found that psychological influences mediated the relationship between institutionally sourced information and vaccine hesitancy (67). Guan et al. found that psychological antecedents played a role in the relationship between community involvement and COVID-19 vaccination behavior played a significant mediating role (45). Zhou et al. validated that fear of COVID-19, as a component of complacency, would mediate the relationship between social media information and vaccine hesitancy (68). Concerning these studies and considering data availability, the present study also chose three psychological antecedents of vaccination, namely self-confidence, complacency, and collective responsibility, as mediating variables to analyze the effect of education on vaccination, where the strategies of the three psychological antecedents of vaccination are detailed in Appendix 1.

Based on these analyses, this study used education as the independent variable, self-confidence, complacency, and collective responsibility as the mediating variables, and vaccination as the dependent variable. AMOS 25.0 was utilized to set up the basic mediator model with single-step multiple, and a fitness test was conducted to correct the basic model based on the fit results. Table 8 shows the fit indices of the model, where the χ^2^ of fitness is 6.159, corresponding to a *p* value greater than 0.05, indicating that the modified structural equation model fits the sample data well. In addition, the RMSEA value is 0.020, and the CFI, GFI, and AGFI values all exceed 0.90, suggesting that the model fits well across all key indicators and can be reliably used for path estimation.

**Table 8:** Model fit indices.

Evaluation indicators	Model result	Adaptation standards	Fitness judgment
χ^2^/Degree of freedom	2.053	<3.00	Yes
χ^2^ probability value	0.104	>0.05	Yes
RMSEA	0.020	<0.08	Yes
CFI	0.992	>0.90	Yes
GFI	0.999	>0.90	Yes
AGFI	0.995	>0.90	Yes
NFI	0.985	>0.90	Yes

#### 3.4.2 Logic structure between education and vaccination

**Figure 8:**
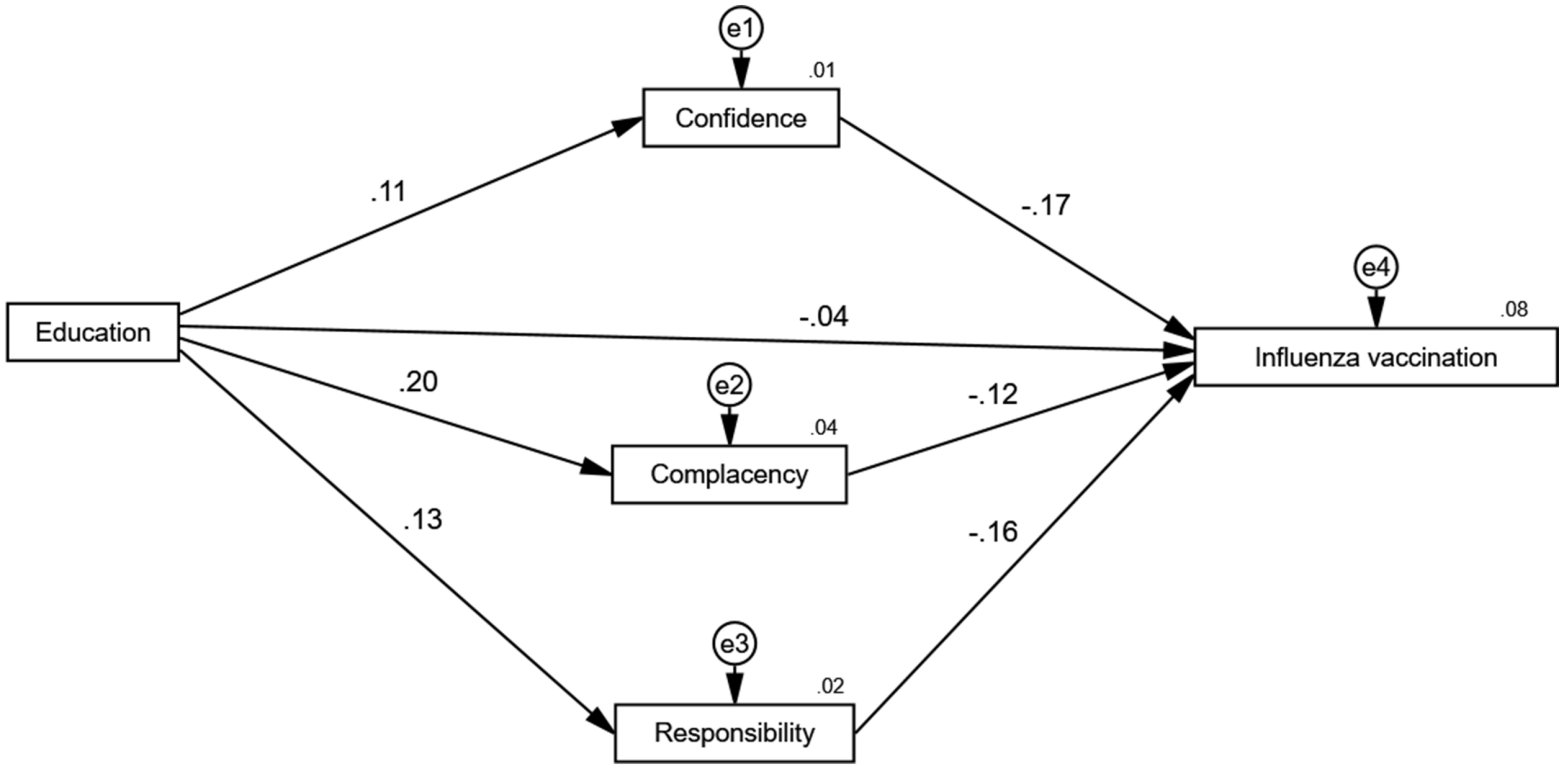
Mediating pathways through which education influences influenza vaccination.

Next, is the estimation of the coefficients of the mediated paths. The Bootstrap method is applied with 5,000 resamples and 95% confidence intervals. Standardized regression coefficients are used as the basis for interpretation. Since AMOS25 does not display significance levels for standardized results, unstandardized significance levels were used to indicate overall significance. Figure 8 demonstrates the path of action of education level in influencing vaccination, while Table 9 specifically presents the regression results with self-confidence, complacency, and collective responsibility as mediators. These results show that education has a significant negative direct effect on respondents’ vaccination with a coefficient size of −0.042, while it has a significant positive effect on self-confidence with a coefficient size of 0.107 at the 1% level of test, a significant positive effect on complacency with a coefficient size of 0.199 at the 1% level of test, a significant positive effect on collective responsibility with a coefficient size of 0.128 at the 1% level of test, while self-confidence and collective responsibility are significantly affected by vaccination. The size of the coefficient is 0.128; while self-confidence, collective responsibility and complacency have a significant negative effect on vaccine hesitancy at the 1% test level, with coefficients of −0.165, −0.125, and −0.162, respectively. These findings support the presence of indirect effects, suggesting that the influence of education on vaccination is partially mediated by these three psychological constructs.

**Table 9:** Results of the mediated pathway test for the influence of education on vaccination.

Paths	Non-standardized coefficient	Standardized coefficient	SE	CR	*p*
Education → Influenza vaccination	−0.02	−0.042	0.009	−2.202	<0.05
Education → Self-confidence	0.231	0.107	0.041	5.562	<0.001
Self-confidence → Influenza vaccination	−0.036	−0.165	0.004	−8.825	<0.001
Education → Self-complacency	0.176	0.199	0.017	10.477	<0.001
Self-complacency → Influenza vaccination	−0.066	−0.125	0.01	−6.572	<0.001
Education → Collective responsibility	0.339	0.128	0.051	6.65	<0.001
Collective responsibility → Influenza vaccination	−0.029	−0.162	0.003	−8.612	<0.001

#### 3.4.3 Results of the mediation effect test of education affecting vaccination

To analyze the mechanism of education level affecting vaccine hesitancy, this study further decomposed its specific pathways. The results of the relevant pathway decomposition are shown in Table 10. It can be seen that, in the relationship between education and vaccination, the indirect effect of self-confidence accounts for 30% of the total effect, the indirect effect of complacency accounts for 37.31%, and the indirect effect of collective responsibility accounts for 3.33%. The 95% CIs of the total, direct, and indirect effects of the three mediating variables did not contain 0, indicating that the mediating effects were significant, i.e., self-confidence, complacency, and collective responsibility mediated the relationship between different types of trust and vaccine hesitation.

**Table 10:** Results of the decomposition of the mediating effect of education on vaccination.

Paths	c	a	b	a*b	a*b		c’	Type of intermediation	Percentage of intermediary effect size
	Total effect			Intermediary effect	(95% BOOT)		Direct effect		
					Lower	Upper			
Education → Confidence → Influenza vaccination	0.06**	0.107**	−0.165**	−0.018	−0.026	−0.011	−0.042*	Part of an intermediary	30.00%
Education → Complacency → Influenza vaccination	0.067**	0.199**	−0.125**	−0.025	−0.035	−0.017	−0.042*	Part of an intermediary	37.31%
Education → Responsibility → Influenza vaccination	0.063**	0.128**	−0.162**	−0.021	−0.029	−0.014	−0.042*	Part of an intermediary	33.33%

**indicates significant at the 5% level of significance; * indicates significant at the 10% level of significance.

## 4 Discussion

To the best of our knowledge, this is the first time that a large Chinese micro-survey database has been used to analyze the key factors influencing influenza vaccination among adults and its equity. Our results show that the influenza vaccination rate among Chinese adults is low, with only 6.75% of adults aged 18 years or older. It is not only lower than the influenza vaccination rate among Chinese children (69), but also lower than the influenza vaccination rate among adults in other countries (70). Furthermore, this rate is also significantly lower than that of other vaccines among Chinese adults, such as the related study that showed the hepatitis B vaccination rate was 14.70% (71). For example, a related study showed that the vaccination rate of hepatitis B among Chinese adults was 14.70% (71). These comparisons underscore the urgent need to strengthen promotional efforts and reduce influenza vaccine hesitancy among the adult population in China.

Regarding the influencing factors of influenza vaccination among adults, similar to the results of previous studies (32, 72, 73), the results of the present study also showed that the influencing factors of vaccination included individuals’ socioeconomic characteristics, health disease status, health insurance, and trust. However, unlike existing studies, this study not only further used the random forest algorithm to identify the key factors influencing influenza vaccination among adults, but also deeply explored the effects of the interaction of different characteristic variables on vaccination. These results are more helpful for targeting and improving the influenza vaccination policy for adults. Among the key influencing factors, the results of this study indicate that there is a negative relationship between the degree of aging concern and adult influenza vaccination, which may be attributed to the lack of awareness of influenza vaccine, lack of confidence in influenza vaccine, and the imperfect preventive vaccination system for adults in China. According to the results of related studies, the current status of influenza vaccination in China is that there is less publicity, vaccination has not been included in the national immunization program, and medical personnel have low willingness to recommend the vaccine due to insufficient incentives (74–76). These systemic barriers hinder knowledge dissemination and weaken respondents’ willingness to be vaccinated. At present, China has entered the stage of deep aging, and the results of this study also show that adults have a high level of concern about aging, and they worry that they will not be able to take care of themselves when they grow old. As vaccination is the most important public health intervention for disease prevention, insufficient influenza vaccination among adults may lead to a more severe disease burden due to autoimmune aging and may also increase the burden on the healthcare system.

Income is an important factor influencing vaccination. The results showed a positive relationship between income level and influenza vaccination, a trend consistent with international evidence. For example, a study in the United States found that each 1% increase in household expenditure on preventive care corresponded to a 4.7% increase in influenza vaccination likelihood (77). The same study from the United States also showed that the proportion of families with an annual income of more than $50,000 who purchased vaccine appointment services (34%) was seven times higher than that of low-income families (78). Similarly, a 2021 report from the German Institute for Employment Research (IAB) found that the average time cost of vaccination for individuals earning ≥30 euros per hour accounted for only 0.58% of daily income, compared to 3.76% for those earning ≤10 euros per hour (79). Currently, influenza vaccine is only included in health insurance coverage in some provinces in China. Government subsidies for influenza vaccination costs are still not implemented in most regions, which increases the cost burden of vaccine recipients, and some adults who need the vaccination may give up the vaccination due to insufficient ability to pay.

In terms of educational attainment, this study showed a negative relationship between education and influenza vaccination among adults. It is not only consistent with the results of existing studies but also different from the results of related studies. In terms of consistency, the results of a French study showed that academics in the humanities and social sciences were 2.1 times more skeptical of institutional medical advice than the general population (80), and “vaccine hesitancy among the tech elite” was found in the Silicon Valley group of high-profile individuals in the United States (81). This finding is also supported by the results of studies from China. Liu et al. analyzed the current status of parental hesitancy to vaccinate children against varicella in some regions of China in 2019. It is found that the proportion of hesitancy was higher among parents with a high level of educational attainment, and the proportion of vaccine hesitancy among parents with a master’s degree or higher was high (82). In terms of variability, Shi et al.’s study, on the other hand, showed that highly educated residents had a high level of knowledge about pandemic prevention, were more concerned about the impact of pandemics on their health, and had a greater willingness to receive the influenza vaccine (83). Klüwer et al.’s findings showed that higher educational attainment was positively correlated with confidence in influenza vaccination (84). The present study suggests that the observed negative relationship may be due to highly educated individuals possessing stronger information-seeking and critical thinking skills, leading to overconfidence in personal judgment. Such individuals may place less trust in public health guidance and become more susceptible to anti-vaccine misinformation, thereby reducing vaccination uptake. Additionally, increased access to vaccine-related information may expose them to more negative content, intensifying their concerns about side effects and decreasing their trust in vaccines. The study further suggests that education may influence vaccination indirectly through psychological factors such as self-confidence, complacency, and collective responsibility.

In terms of age, this study showed a negative relationship between age and influenza vaccination, a finding supported by prior research. The results of related studies showed that the main reasons for the lower vaccination willingness of older adult individuals included fear of vaccine side effects, poor vaccination accessibility, insufficient willingness of medical personnel to recommend, and the adverse effects of peer communication (28, 32, 85, 86). For example, some studies have suggested that some older adult individuals suffer from underlying diseases and a weaker immune system, which makes them wary of vaccinations and believe that they do not get better results (87, 88). It has also been suggested that older adult individuals may face difficulties such as poor transportation, ill health, and long distances that make it difficult for them to conveniently travel to the vaccination site for vaccination, resulting in fewer opportunities to be vaccinated (89). In addition, some older adult individuals may not be recommended by healthcare professionals or fail to receive advice or reminders to get vaccinated from their physicians, and thus will not be motivated to get vaccinated against influenza (90, 91).

The result that health insurance can hurt influenza vaccination in adults is at variance with previous relevant studies, such as those from the United States. It showed that vaccination rates were significantly lower in the uninsured or underinsured population than in the insured population. Adult influenza vaccination rates were 44.3% among the insured compared to only 14.4% among the uninsured (92). This study suggests that this difference arises because health insurance coverage policies for vaccination are different. In China, basic health insurance is mainly used for disease treatment, and preventive vaccines are usually not covered. According to China’s Social Insurance Law, preventive vaccines are not covered by health insurance, and disease prevention and control programs, such as vaccines and vaccination costs, should be addressed through public health service funding channels. Therefore, the types of vaccines covered by health insurance are relatively limited, and the influenza vaccine also falls into the category of non-immunization planning. Influenza vaccination is not included in the reimbursement scope of basic medical insurance, resulting in the need for individuals to pay for the vaccination at their own expense, which reduces the willingness to be vaccinated. Although some regions in China have introduced subsidies, such as allowing surplus health insurance funds to offset out-of-pocket vaccine costs, barriers still persist. These include the uneven distribution of vaccination clinics, difficulty in securing appointments, and transportation challenges, all of which negatively affect the accessibility and convenience of vaccination services and, ultimately, vaccination uptake.

Trust is also an important factor influencing vaccination. Numerous studies conducted in China and other countries have explored the relationship between trust and vaccination. The findings suggest that trust in the government and doctors increases vaccine confidence and reduces vaccine hesitancy (93, 94). The results of this study are also consistent with previous related studies that government trust and physician trust positively and positively affect influenza vaccination in adults. This suggests that increasing the level of trust as an intervention may help prevent and mitigate vaccine hesitancy and increase vaccination, especially in the context of the numerous infectious disease threats facing society today. As a policymaker, the government plays an important role in the prevention and control of epidemics, and increased levels of trust in the government can help people implement and comply with government policies during emergencies such as epidemics. Doctors can play an important role in mitigating vaccine hesitancy. Doctors can use their expertise to communicate the value of vaccination to the public and increase people’s awareness of vaccine safety and efficacy. However, the results of a related study on vaccine hesitancy in China showed that physicians were not highly motivated to recommend vaccines to patients in clinical practice, and the positive role of physicians in reducing vaccine hesitancy has not been effectively played (95). Therefore, further reduction of vaccine hesitancy should focus on improving the professional role of physicians.

Consistent with previous related studies (96, 97), the present findings also reveal a negative relationship between health status and vaccination. This phenomenon may be due to the misalignment between individuals’ subjective perception of their health status and their behavioral decision-making. Some adults believe that they are healthy, have good immunity, are not susceptible to influenza, and can recover quickly even if they are infected. This “health illusion” leads them to underestimate the severity of influenza and the need for vaccination, thus reducing their willingness to be vaccinated. Some adults are also concerned about the side effects of the vaccine. They are worried that the influenza vaccination may cause side effects such as fever and muscle pains, and believe that these discomforts will affect their daily life and work. Therefore, they may choose not to get vaccinated to avoid possible discomfort. Finally, some people do not have sufficient knowledge about the mechanism of action, effectiveness, and safety of the influenza vaccine, or are influenced by misinformation and mistakenly believe that the vaccine is ineffective or unsafe, and this lack of information and misunderstanding also reduces their willingness to be vaccinated.

In terms of influencing factors, this study further analyzed the effects of interactions between different characteristic variables on influenza vaccination among adults. The relationship between these interaction terms and vaccination expands the influence of single factors on vaccination and helps to analyze the complex influencing mechanisms of vaccination at a deeper level. The findings suggest that interactions between different factors can have differential effects on influenza vaccination, with positive associations between some of the interaction terms and the target variables and negative associations between other interaction terms and vaccination. These findings underscore both the complexity of the factors influencing adult vaccination and the need to address vaccine hesitancy through comprehensive, multifactorial intervention strategies.

In terms of vaccination equity, the results of this study show that influenza vaccination behavior among adults is concentrated in the high-income group. This is consistent with previous research on vaccination equity in China (41). The cost of vaccination is an important factor influencing vaccination behavior; in China, the influenza vaccine has not yet been included in the national immunization program, and adults in most areas need to pay for influenza vaccination themselves. For adults with higher income levels, their ability to pay is stronger, their sensitivity to vaccine prices is low, and their own vaccination needs are easily satisfied. For lower-income groups, on the other hand, they may forgo vaccination due to insufficient ability to pay, at which point cost becomes a barrier factor between willingness to vaccinate and vaccination behavior. In addition, our findings show that age, income, education, and aging concerns are important factors contributing to inequities in influenza vaccination among adults.

## 5 Policy recommendations

Based on our findings, the following recommendations are proposed. First, the role of trust in reducing vaccine hesitancy and increasing vaccination is emphasized. This study proves that both government trust and physician trust help promote vaccination, which means that further improvement of the adult influenza vaccination rate needs to emphasize the role of trust. During epidemics of infectious diseases, the government should focus on releasing authoritative news, communicating prevention and control policies to the public promptly, and maintaining communication with the public. At the same time, relevant government professional organizations (e.g., CDC, etc.) can release timely data on the benefits of vaccination, use real-world data to eliminate public concerns about vaccine side effects, and boost confidence in vaccination. Doctors can also play an important role in increasing vaccination rates by explaining the safety and effectiveness of vaccines and the need for vaccination through face-to-face counseling, written information, and other media during patient visits. They can also provide patients with personalized vaccination recommendations based on the patient’s health status, age, and occupational characteristics, with the “Vaccine Prescription,” which is currently being piloted in several provinces, being implemented. It is a useful attempt for doctors to actively participate in vaccination recommendations. In addition, doctors can also disseminate scientific vaccine knowledge to a wider range of people and oppose vaccine-related misinformation and rumors through community activities, health lectures, and social media platforms. Second, subsidize the cost of influenza vaccination to reduce the burden of vaccination on adults. Currently, the influenza vaccine has not been included in the scope of the national immunization plan, which will increase the cost burden of the vaccinated and is not conducive to the increase of the vaccination rate. The government can consider subsidizing the cost of vaccination, such as including influenza vaccination in the public health program or the health insurance payment catalog. The Affordable Care Act in the United States stipulates that insurance companies must cover the cost of recommended vaccinations and may not charge insured persons co-payments or out-of-pocket expenses. In addition, the government can also learn from the centralized band purchasing policy for medicines implemented in China and consider centralized purchasing for vaccines. This approach would enhance institutional bargaining power and reduce both purchase and administration costs, ultimately making vaccination more accessible. Third, focus on special populations to promote fairness in vaccination. In addition to low overall vaccination rates, disparities in access to influenza vaccination persist. Special attention should be directed toward vulnerable populations such as low-income individuals and the older adult. The government should allocate additional public health resources to these groups to ensure equitable access. For example, for low-income and older adult groups, the government can subsidize influenza vaccines or directly provide free vaccination services through medical insurance and social assistance, which can effectively reduce their economic burden and improve vaccination willingness. It can also provide free vaccination services at community health service centers. This can effectively reduce their financial burden and increase their willingness to be vaccinated. The government can also increase the number of vaccination points in community health service centers and activity centers for the older adult, or provide door-to-door vaccination services for older adult people with mobility difficulties, to improve the convenience of vaccination. Fourth, according to the interaction of multiple factors, the role of comprehensive intervention strategies should be fully utilized. Adult influenza vaccination is affected by the interaction of different factors. Therefore, comprehensive intervention strategies should be emphasized to further increase the vaccination rate. Currently, information reminders, education and training, and material incentives have been proven to be effective but remain underutilized in practice. Implementation science offers a promising approach to integrate various interventions into a cohesive strategy, addressing the limitations of isolated measures and accelerating their application. In future public health initiatives, incorporating implementation science frameworks can enhance the effectiveness and scalability of vaccination interventions, ultimately increasing adult vaccination rates.

## 6 Conclusion

Our findings suggest low influenza vaccination rates among Chinese adults, as well as inequities in influenza vaccination among adults. These results are influenced by multiple factors, including educational attainment, aging concerns, income, age, physician trust, and health insurance. The findings have important practical implications: by providing new evidence on influenza vaccination among Chinese adults, this study contributes to the optimization of adult preventive immunization policies. To further increase influenza vaccination rates as well as promote vaccination equity, future optimization of adult immunization policies should emphasize valuing the role of trust in improving vaccination, subsidizing the cost of vaccination, and making full use of comprehensive intervention strategies in order to promote influenza vaccination and further promote vaccination equity.

## 7 Limitations

Although this study provides results on the key influencing factors of influenza vaccination and its equity among Chinese adults, there are still some limitations that need to be addressed. First, the data are derived from secondary data from a publicly available dataset (the most recent version of this database was published in 2021), and the data has a certain lag. Future analyses using more recent data are needed to capture evolving vaccination trends and determinants. Second, the study is based on cross-sectional survey data, which restricts the ability to infer causal relationships between influenza vaccination and its influencing factors. Longitudinal data in future studies could enable better analysis of temporal dynamics and causal pathways. Third, while the CGSS is nationally representative, it still has limitations, such as respondents’ subjectivity in answering the questionnaire. The vaccinations utilized in this study were self-reported and may have recall bias. Fourth, although the study included a wide range of explanatory variables, certain influential factors such as attitudes toward vaccine manufacturers or sources of vaccine information were not captured due to the absence of appropriate proxy variables in the dataset. Future studies can incorporate such factors as more comprehensive data become available. Fifth, the study analyzed the influencing factors of influenza vaccination among adults using the random forest model and logistic regression method, and the combination of the two methods helps to overcome the shortcomings of a single method. However, other machine learning techniques such as decision trees and XGBoost were not utilized. Future studies could incorporate these methods to strengthen the robustness of findings and provide more nuanced evidence. Sixth, the relatively limited sample size, constrained by the cross-sectional design, may reduce the statistical power and generalizability of the results. This limitation could affect the ability to detect significant associations or to generalize findings to a broader population. Future research can benefit from larger and more diverse longitudinal datasets and may consider alternative study designs or complementary statistical approaches to enhance the reliability and external validity of the findings.

## Data Availability

Publicly available datasets were analyzed in this study. This study was based on a publicly available database. Dataset/questionnaire/interview used in our study has previously been published elsewhere, this information is available through the China Survey and Data Center at Renmin University of China. The datasets generated and/or analyzed during the current study can be found at: http://cgss.ruc.edu.cn/.
